# *Helicobacter pylori* Type IV Secretion Apparatus Exploits β1 Integrin in a Novel RGD-Independent Manner

**DOI:** 10.1371/journal.ppat.1000684

**Published:** 2009-12-04

**Authors:** Luisa F. Jiménez-Soto, Stefan Kutter, Xaver Sewald, Claudia Ertl, Evelyn Weiss, Ulrike Kapp, Manfred Rohde, Torsten Pirch, Kirsten Jung, S. Francesco Retta, Laurent Terradot, Wolfgang Fischer, Rainer Haas

**Affiliations:** 1 Max von Pettenkofer-Institute for Hygiene and Medical Microbiology, Ludwig-Maximilians-Universität, München, Germany; 2 Macromolecular Crystallography Group, European Synchrotron Radiation Facility, Grenoble, France; 3 Helmholtz Center for Infection Research, Department of Microbial Pathogenesis, Braunschweig, Germany; 4 Munich Center of integrated Protein Science, CiPSM, at the Department of Biology, Microbiology, of the Ludwig-Maximilians-Universität München, Planegg-Martinsried, Germany; 5 Molecular Biotechnology Centre, Department of Genetics, Biology and Biochemistry, Torino, Italy; Institut Pasteur, France

## Abstract

Translocation of the *Helicobacter pylori* (*Hp*) cytotoxin-associated gene A (CagA) effector protein via the *cag*-Type IV Secretion System (T4SS) into host cells is a major risk factor for severe gastric diseases, including gastric cancer. However, the mechanism of translocation and the requirements from the host cell for that event are not well understood. The T4SS consists of inner- and outer membrane-spanning Cag protein complexes and a surface-located pilus. Previously an arginine-glycine-aspartate (RGD)-dependent typical integrin/ligand type interaction of CagL with α5β1 integrin was reported to be essential for CagA translocation. Here we report a specific binding of the T4SS-pilus-associated components CagY and the effector protein CagA to the host cell β1 Integrin receptor. Surface plasmon resonance measurements revealed that CagA binding to α5β1 integrin is rather strong (dissociation constant, K_D_ of 0.15 nM), in comparison to the reported RGD-dependent integrin/fibronectin interaction (K_D_ of 15 nM). For CagA translocation the extracellular part of the β1 integrin subunit is necessary, but not its cytoplasmic domain, nor downstream signalling via integrin-linked kinase. A set of β1 integrin-specific monoclonal antibodies directed against various defined β1 integrin epitopes, such as the PSI, the I-like, the EGF or the β-tail domain, were unable to interfere with CagA translocation. However, a specific antibody (9EG7), which stabilises the open active conformation of β1 integrin heterodimers, efficiently blocked CagA translocation. Our data support a novel model in which the *cag*-T4SS exploits the β1 integrin receptor by an RGD-independent interaction that involves a conformational switch from the open (extended) to the closed (bent) conformation, to initiate effector protein translocation.

## Author Summary

Integrins are single transmembrane proteins present on almost all types of cells. They are composed of an α and a β subunit, which together form the ligand binding pocket, able to interact with extracellular matrix proteins. The best known binding domain on integrin ligands is the RGD domain. Many bacterial, but also viral pathogens exploit this ligand-binding domain to interact with integrins on the host cell. *Helicobacter pylori*, a common bacterial pathogen associated with gastric diseases, was recently added to this list. One of *H. pylori's* most important factors associated with gastric pathologies is the CagA protein. This protein is directly injected into host cells through the Cag Type IV Secretion System (*cag*-T4SS). Previous studies demonstrated that the *cag*-T4SS requires integrins for the injection (translocation) of CagA into cells. We provide evidence that three proteins, CagA, CagI and CagY, interact with integrins in an RGD-independent way. Additionally, our data point out that the Cag apparatus needs the physical capacity of a β1 integrin heterodimer to change from an active/extended conformation to a closed/bent conformation. This novel kind of integrin interaction opens a new way in which pathogens can use receptors on cells.

## Introduction

Infection with the gastric pathogen *Helicobacter pylori* (*Hp*) is associated with a spectrum of pathologies, ranging from mild gastritis to peptic ulcers and gastric cancer [1]. However, the molecular mechanisms underlying the development of *Hp*-associated gastroduodenal diseases are still poorly defined. Two major virulence factors of *Hp* that have been associated with disease induction are the vacuolating cytotoxin (VacA) and the cytotoxin-associated antigen A (CagA), both of which are delivered into eukaryotic target cells. VacA, a secreted multifunctional protein toxin, induces intracellular vacuoles in epithelial cells, inhibits T lymphocyte proliferation and modulates T cell function [reviewed in 2]. Using the β2 integrin subunit CD18 as a cellular receptor for uptake [3], VacA efficiently down-regulates transcription of several cytokines or chemokines in T cells [4]. CagA, an immunodominant protein of 120–170 kDa, is encoded on the *cag* pathogenicity island (*cag*-PAI). The *cag*-PAI comprises a total of 27 genes, encoding the *cag*-Type IV Secretion System (T4SS) in *Hp* [5].

Upon direct contact with gastric epithelial cells, CagA is translocated into host cells via the *cag*-T4SS [6] and immediately tyrosine-phosphorylated at a variable number of so-called EPIYA motifs by kinases of the Src and c-Abl family [7],[8]. CagA interacts with a large set of host proteins in phosphorylation-dependent and -independent ways and is considered as a bacterial oncoprotein that exerts multiple effects on host signal transduction pathways, the cytoskeleton and cellular junctions [reviewed in 9]. A further hallmark of *cag*-PAI positive *Hp* strains is their ability to induce the secretion of chemokines upon contact with epithelial cells, such as interleukin-8 (IL-8) [10]. The function of each *cag*-PAI-encoded component for CagA delivery and IL-8 secretion has been studied by a systematic mutagenesis approach [11]. Translocation-competent *Hp* strains harbour membrane protrusions consisting of a central filament, carrying on its surface the *cag*-PAI encoded proteins CagY (HP0527), a VirB10 homologous protein [12], CagX (HP0528, VirB9-homologue) and CagT (HP0532, VirB7-homologue) [13]. Although the ultrastructure and the biochemical composition of these protrusions has not been clarified yet, we would refer to these structures as type IV secretion system pili. Furthermore, the α5β1 integrin heterodimer has recently been identified as a receptor for the *Hp* pilus-associated adhesin CagL [14].

Integrins represent a family of about 24 different αβ heterodimeric receptors that mediate cell-cell, cell-extracellular matrix and cell-pathogen interactions and govern migration and anchorage of almost all kinds of cells. Each of the non-covalently associated subunits contains a large N-terminal extracellular domain, a transmembrane segment and a short C-terminal cytoplasmic tail. Affinity for biological ligands is regulated by inside-out and outside-in signalling. The bent conformation of the integrin heterodimer represents the physiological low-affinity state, whereas inside–out signalling and ligand binding induces a large-scale conformational rearrangement, in which the integrin extends from the bent into an extended, open conformation [15]. *Hp* T4SS-pilus-associated CagL was suggested to bind via its arginine-glycine-aspartate (RGD) motif to α5β1 integrin, a process described as essential for CagA translocation and activation of focal adhesion kinase (FAK) and Src kinase [14]. In the present study we show that further components of the *cag*-T4SS, such as CagY (HP0527) and the effector protein CagA interact with distinct extracellular domains of β1 integrin. These components are located along or at the tip of the T4SS-pilus. We propose a model that suggests conformational changes of the integrin heterodimer as a basis for CagA translocation.

## Results

### CagA Translocation into Epithelial Cells is Dependent on β1 Integrin Heterodimers

**Figure 1:**
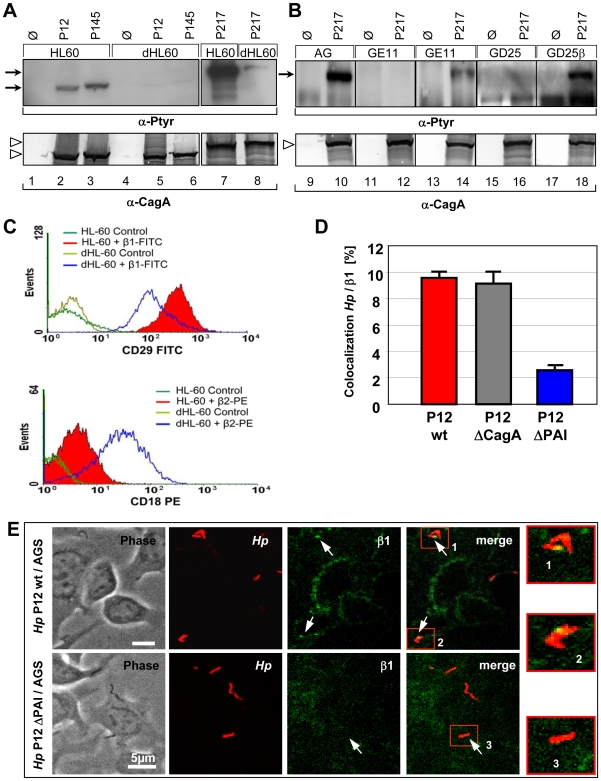
Host cell β1 integrin is essential for *Hp* to translocate and tyrosine-phosphorylate CagA in different cell lines. (A) Immunoblots to determine translocation of CagA in HL60 versus dHL60 cells. (B) Immunoblots of β1 integrin knockout fibroblasts (GD25), epithelial cells (GE11) or β1 gene-complemented cells (GD25β, GE11β) infected with *Hp* strains or media (control). The upper panel shows CagA translocation using a phosphotyrosine-specific antibody (mAb PY99), the lower panel shows CagA production by *Hp*. Bands corresponding to Phospho-CagA (CagA-P) or CagA are indicated by arrows or open arrowheads, respectively. (C) FACS analysis quantifying β1 and β2 integrin surface localization on HL60 and dHL60 cells using antibody CD29 FITC (β1 integrin) or CD18 PE (β2 integrin). (D) Quantification of co-localization events of *Hp* wt, Δ*cag*A or ΔPAI bacteria and β1-integrin by laser scanning confocal microscopy. (E) Confocal micrographs, showing binding of *Hp* P12-*gfp* wt (upper panel) or P12ΔPAI (lower panel) and β1-integrin-labelled AGS cells (4B7-AlexaFluor_568_) and areas of co-localization of *Hp* and β1 integrin (arrows).

*Hp* translocates its effector protein CagA via the *cag*-T4SS into a number of different cell types *in vitro* [16]. Recently it was shown that CagA translocation is dependent on the interaction of the *Hp* T4SS-pilus-associated protein CagL, binding in an RGD-dependent way to α5β1 integrin on the host cell [14]. However, nothing is known about the mechanism of CagA translocation and the involvement of other T4SS components in this process. Using a different approach, we also identified β1 integrin as a cellular receptor for the *cag*-T4SS. Three independent *Hp* strains (P12, P145, P217) were applied to study host cell requirements for CagA translocation using several human and animal cell lines (data not shown). Of special interest were human promyelocytic leukaemia (HL60) cells, which were fully competent for CagA translocation (Figure 1A, lanes 1–3 and 7), whereas, HL60 cells differentiated to a granulocyte-like phenotype (dHL60 cells) revealed only a very weak CagA-P signal (Figure 1A, lanes 4–6 and 8). Thus, the capacity of *Hp* to translocate CagA varies considerably, even for the same type of cell, dependent on its cellular differentiation stage. Flow cytometry revealed elevated β2, but significantly reduced β1 integrin levels on the surface of dHL60, as compared to HL60 cells (Figure 1C). We therefore concentrated on β1 integrin as a potential receptor for the T4SS. CagA translocation was completely absent for epithelial (GE11) or fibroblast-like (GD25) β1 integrin knockout cells, but was functional in genetically complemented GE11β or GD25β cells (Figure 1B) [17]. In agreement with Kwok et al. [14], these data independently confirmed the important finding that β1 integrin is essential for CagA translocation.

### CagA, CagY and CagI are β1 Integrin Interaction Partners

**Figure 2:**
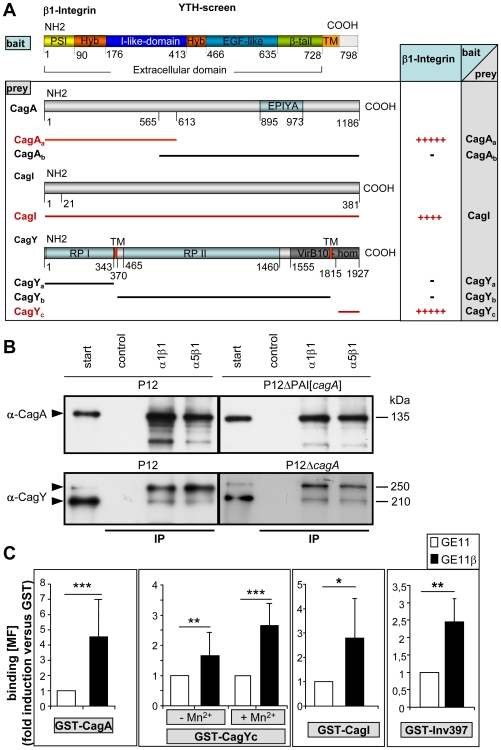
Identification and verification of *Hp* T4SS proteins interacting with human β1 integrin. (A) Domain organization of the β1 integrin clone used as bait in a YTH screen and identification of interacting proteins encoded by the *cag*-PAI used as preys. (+, interaction; −, no interaction), tenfold dilution steps still allowing growth of yeast on selective media are indicated by number of “+” signs (see Figure S1). (B) For pulldown assays, magnetic beads (SiMAG) were loaded with purified functional α1β1 or α5β1 integrin or Tris buffer (control) and incubated with processed *Hp* P12 wt or defined mutant strains, as indicated (fraction Soluble II, Figure S2A). Beads were recovered by magnetic forces, washed, boiled and run for SDS-PAGE. Immunoblotting with α-CagA or α-CagY antibody detects precipitated proteins (arrowheads). (C) Quantification of binding of purified GST-CagA, GST-CagYc, GST-CagI or GST-Inv397 (30µg/1×10^6^ cells) to integrin-deficient GE11 versus integrin-proficient GE11β cells by flow cytometry. Binding is analysed by α-GST antibody (fold increase in binding versus GST, the values for binding of GST alone has been set to 1). RPI, repeat region I, RPII, repeat region II, (*, p<0.05; **, p<0.01; ***, p<0.001; students T-test). MF, mean fluorescence, (n = 20). Oligonucleotides for construction of GST fusion proteins see Table S2.

CagL is the only protein encoded on the *cag*-PAI which carries an RGD motif and therefore might be recognized by the α5β1 integrin receptor in a typical integrin/ligand–like fashion. Whereas Kwok et al. [14] specifically concentrated on the CagL/α5β1 integrin interaction, we chose a systematic approach to identify possible T4SS-integrin interactions and applied a yeast two-hybrid (YTH) screen using the GAL4 Matchmaker system (Clontech) (Figure 2A). Since various cell lines expressing different α/β1 integrin combinations proved successful for CagA translocation (data not shown), the β1 subunit was considered as important and the extracellular portion of the human β1 integrin gene was used as bait. As prey for the YTH screen each of the 27 *cag*-PAI-encoded proteins were assayed [18]. Positive interactions were obtained for the extracellular part of β1 integrin with the N-terminal region of CagA (HP0547_a_), the C-terminal (VirB10-homologous) portion of CagY (HP0527_c_) and with CagI (HP0540) (Figure 2A, Figure S1). Similar results were obtained when bait and prey were exchanged (data not shown).

To confirm the YTH data, pulldown experiments using *Hp* T4SS-associated proteins were performed (see Figure S2A for preparation of extracts) using functional α1β1 and α5β1 integrin heterodimers (Chemicon) coupled to magnetic beads. CagA was specifically pulled down from wt cell lysates by α1β1 or α5β1 integrin beads, but not by controls (Tris-blocked beads) (Figure 2B). The ectopic expression of *cag*A from the shuttle plasmid pJP66 [19] in a P12ΔPAI strain demonstrated that CagA alone is able to interact with β1 integrin without any other component of the *cag*-PAI. Preferentially the upper band of CagY and only small amount of the lower band of CagY was pulled down by the same procedure (Figure 2B, lower panel). Again, precipitation of CagY from a *cag*A-negative *Hp* background confirmed an interaction of CagY with integrin, independent of CagA.

The putative interaction of β1 integrin with CagI could not yet be verified by pulldown assays, due to the lack of a specific functional antibody against CagI. We therefore used as a further method a cell-based assay to determine binding of the corresponding GST-Cag fusion proteins to β1 integrin-proficient (GE11β), versus β1 integrin-deficient cells (GE11) by flow cytometry (Figure 2C and Figure S2B,C). GST did not bind β1–integrin-dependent, but purified GST-CagA, GST-CagI and GST-CagYc bound significantly more efficiently to GE11β as compared to GE11 cells (Figure 2C). Interestingly, GST-CagY, but not the other GST fusion proteins, bound more efficiently when the integrins were activated by Mn^2+^. The *Yersinia* invasin (GST-Inv397) [20], known to specifically bind β1 integrin, showed a similar behaviour in these binding assays as the GST-Cag proteins (Figure 2C). To exclude that these GST fusion proteins would bind unspecifically to the GE11β cells, we also generated unrelated *cag*-PAI GST fusion proteins, such as GST-Cagβ (HP0524), GST-CagG (HP0542) and GST-CagZ (HP0526), which did not show β1 integrin-specific binding to the cells (Figure S2B). These data confirmed the β1 integrin-specific binding of CagI from the YTH assay and verified CagI as an additional *cag*-T4SS component interacting with β1 integrin. Unexpectedly, neither α1β1, nor α5β1 integrin beads specifically pulled down CagL from membrane or soluble fractions of *Hp* wt cells using our precipitation conditions (data not shown). We therefore also generated GST-CagL and GST-CagL-RAD mutant protein. Although both purified proteins revealed a rather weak interaction to β1 integrin, the binding was completely independent from the RGD motif of CagL (Figure S2C).

Taken together, these data verified CagA (the translocated effector protein), CagY and CagI as direct interaction partners of different β1 integrin heterodimers. The fact that β1 integrin in combination with different integrin α chains (α1, α5) precipitated the Cag proteins confirmed that these proteins bind to the β1 subunit, rather than the α subunit of the heterodimer and strongly support the YTH results.

### Localization of CagY and CagA Along the T4SS Pilus or Tip

To allow binding of CagA, CagY and CagI to the integrin receptor, these T4SS components should be accessible at the surface of the T4SS pilus. CagY is known as an essential component of the membrane-spanning T4SS complex, but in addition its surface- or T4SS pilus-association has also been demonstrated [12],[13]. In addition to CagY, we also verified CagA on the pilus by field emission scanning electron microscopy (FESEM) (Protocol S1) (Figure S3) [12]. Anti-CagA-coupled gold particles preferentially labelled the pilus tip, with one or rarely two gold grains only, but no staining of the pilus base and only rare background staining of the bacterial or eukaryotic cell surfaces was visible (Figure S3A-I). To investigate a binding of the *cag*-T4SS pilus to β1 Integrin during the infection process, confocal laser scanning microscopy (CLSM) and life cell imaging were applied. The *gfp*-expressing *Hp* P12 wild type (wt) strain, but not the equally well binding P12ΔPAI mutant strain, showed a rapid co-localization with β1 Integrin upon infection of AGS cells (Figure 1E). Quantification data revealed that roughly 10% of *Hp* P12 wt or P12Δ*cag*A, but only 2.5% of P12ΔPAI bacteria co-localized with β1 Integrin (Figure 1D). A P12Δ*cag*A deletion mutant still showed co-localization to β1 integrin, which can be explained by its binding via CagY or CagI.

### The RGD Motif in CagL is not Essential for T4SS Function

**Figure 3:**
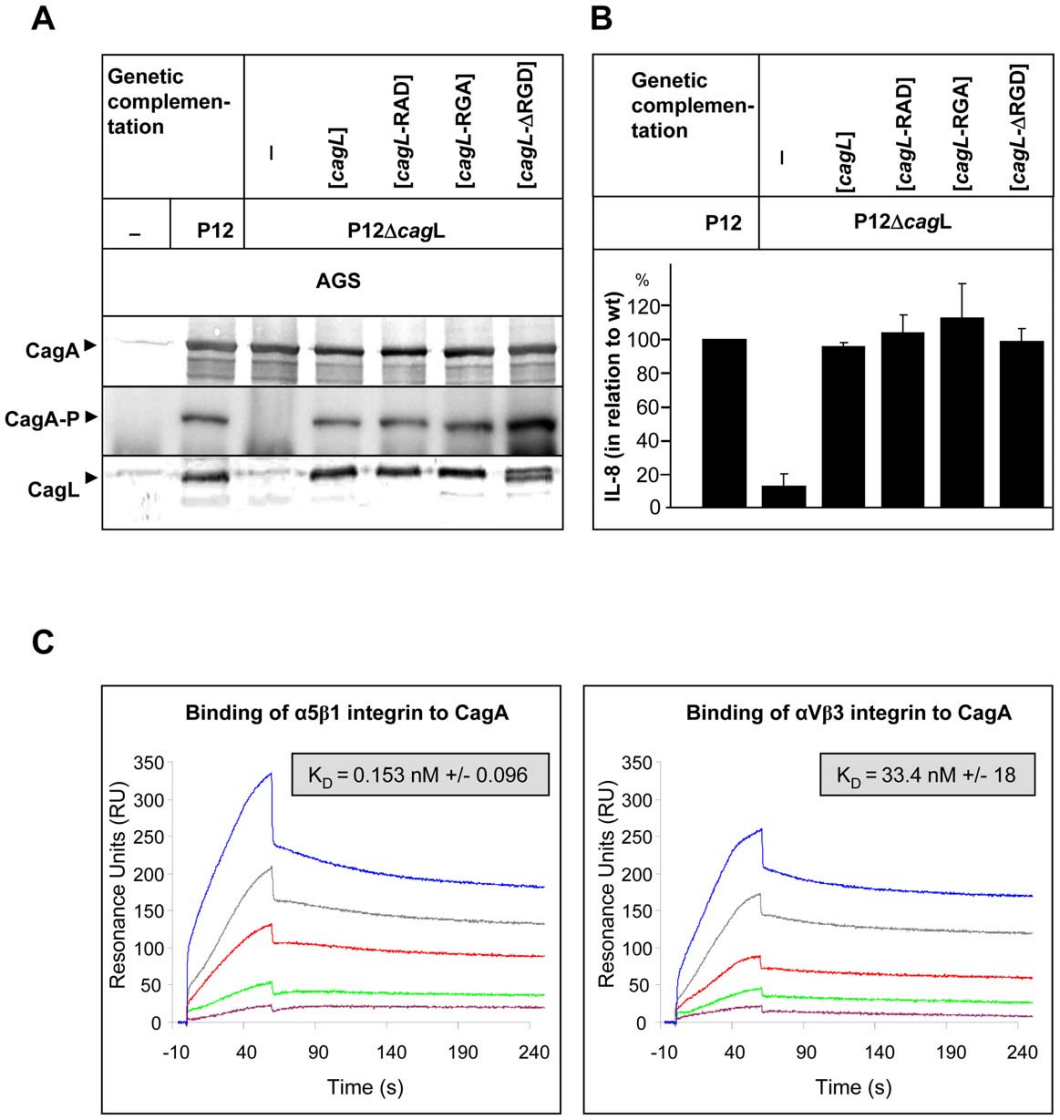
Construction and functional characterization of *Hp* P12 strains producing CagL proteins with defined amino acid exchanges in the RGD motif and CagA binding to β1 integrin. (A) Immunoblot of P12 wt and P12Δ*cagL* strains genetically complemented by a *cagL* wt or a mutated gene carrying a RAD or a RGA exchange, or an RGD empty site (ΔRGD). (B) Sandwich ELISA to determine the IL-8 secretion of AGS cells infected by P12 wt or specific *cagL* mutant strains. (C) Surface plasmon resonance sensograms of the interaction of immobilized CagA (stable 100 kDa N-terminal fragment) with integrin α5β1 (left) and αVβ3 (right) (0.07–1.12 ng/µl) shown in resonance units (RU). The following concentrations of integrin were used: magenta, 0.07 ng/µl; green, 0.14 ng/µl; red, 0.28 ng/µl; grey, 0.56 ng/µl; blue, 1.12 ng/µl. (n = 10 individual measurements and K_D_ calculations).

We wondered why CagL was detected neither in our YTH screen, nor the pulldown assays. To reassess the described RGD-dependent interaction of CagL and α5β1 integrin [14], we first generated a defined *cagL* deletion mutant in *Hp* P12. The strain was genetically complemented in the *Hp recA* locus by mutated *cagL* genes encoding RAD, RGA or ΔRGD versions of CagL (Figure 3A). AGS cells infected with the P12Δ*cagL* strain failed to translocate CagA and to induce IL-8, as described earlier [11],[14], but surprisingly all three distinct *Hp cagL* mutant strains (*cagL*-RAD, *cagL*-RGA and *cagL*ΔRGD) behaved identical to the P12 wt strain concerning CagA translocation, as well as IL-8 induction (Figure 3B). This clearly indicated that under infection conditions an RGD-mediated interaction of CagL with α5β1 integrin either is not existent, or not necessary for CagA translocation, or for IL-8 induction.

### Binding of CagA to β1 Integrin with High Affinity

To judge the specificity and the strength of CagA binding to β1 integrin, surface plasmon resonance measurements were performed. The expression of recombinant CagA is problematic because the protein is rapidly degraded [21],[22]. We considered this property as we purified a stable N-terminal fragment (100 kDa) of CagA, lacking the C-terminal 33kDa domain [22]. The protein was used for binding studies with purified α5β1 integrin and αVβ3 integrin (Clontech) as a negative control. CagA binds with high affinity to α5β1 integrin (K_D_ = 0.153+/−0.096 nM) (Figure 3C) and with a 2-log higher K_D_ value to αVβ3 integrin (K_D_ = 33.4+/−18 nM) (Figure 3D), demonstrating the avidity and the specificity of CagA for β1 integrin binding. Thus, the K_D_ value of α5β1 and CagA is approximately hundredfold lower as compared to the same integrin with its cognate ligand, fibronectin, which is dependent on an RGD motif (K_D_ = 15 nM) [23]. This strong and specific binding suggests an important function for this interaction.

### Integrin Clustering is Essential for CagA Translocation, but not Signalling via the β1 Integrin Cytoplasmic Tail

Specific binding of CagA or CagY to the β1 integrin subunit of the heterodimer on the host cell surface might stimulate integrin clustering and internalization. Cholesterol depletion of AGS cells by methyl-β-cyclodextrin strongly reduced CagA translocation in a dose and time-dependent manner (Table 1). Calpeptin inhibits the Ca^2+^-dependent protease calpain, which is required for the release of integrins from the cytoskeleton and for clustering in lipid rafts. Calpeptin treatment completely abrogated CagA translocation (Figure S4, Table 1) and together with the methyl-β-cyclodextrin data strongly suggested that the organization of β1 integrins into lipid rafts and integrin clustering is essential for CagA translocation, as recently confirmed [24].

**Table 1:** Ligands or inhibitors of integrin signalling and their effect on CagA translocation.

Treatment	Concentration	Target	CagA Translocation	Ref
None	-	-	**+++**	**-**
**Integrin activating and **				**-**
**inactivation Ions**				**this study**
CaCl2	10 mM	Extracellular inhibitor of Integrin, binding of fibronectin	**+++**	**this study**
MnCl2	2 mM	Extracellular activator of Integrin, binding of fibronectin	**++++**	**this study**
EDTA	0.8–1.2 mM	Extracellular divalent cation chelator (Ca^2+^ & Mg^2+^)	**+++**	**this study**
BAPTA	60 µM	Intracellular calcium chelator	**+**	**this study**
**Proteases, Integrin ligands, antibodies**				**-**
8E3	30 µg/ml	Stimulatory mab, binds inactive form of the β1 integrin PSI domain	**+++** 1	**[40]**
N29	30 µg/ml	Stimulatory mab, binds β1 integrin PSI domain	**+++** 1	**[41]**
JB1A	30 µg/ml	Inhibitory mab, binds β1 integrin PSI domain	**+++** 1	**[42]**
AIIB2	30 µg/ml	Inhibitory mab, inactivates β1 integrin binding to substrate	**+++** 1	**[43]**
12G10	30 µg/ml	Stimulatory/inhibitory mab, binds to active form of β1	**+++** 1	**[44]**
K20	30 µg/ml	Neutral mab, binds to hybrid/EGF-like region of β1	**+++** 1	**[45]**
LM534	30 µg/ml	Neutral mab, binds to hybrid/EGF-like/β-tail region of β1	**+++** 1	**[46]**
B3B11	30 µg/ml	Stimulatory mab, binds to β-tail region of β1	**+++** 1	**[41]**
9EG7	30 µg/ml	Stimulatory/inhibitory mab, binds EGF-like region of activated β1	**−** 1	**[33]**
Trypsin	10 µg/ml	Digestion of extracellular proteins	**++++**	**this study**
Thrombin	4 U/ml	Digestion of extracellular proteins	**++++**	**this study**
RGD peptide	5–100µg/ml	Binding to vWF domain of integrin Competition of binding to fibronectin	**+++** 1	**this study**
RAD peptide	5–100µg/ml	Peptide control of RGD	**+++** 1	**this study**
Invasin 197 & 397	5 µg/ml	Binding to β1 integrin (CD29), induces uptake	**+++** 1	**this study**
Fibronectin	100–10 µg/ml	Ligand of α5β1 integrin	**+++** 1	**this study**
**Inhibitors membrane signaling & lipid raft formation**				**-**
Methyl-β-cyclodextrin	0.1 g/ml	Cholesterol depletion from membranes. Lipid rafts disturbance	**(+)** 2	**this study**
Calpeptin	280 µM	Serine/threonine protease inhibitor, inhibits calpain proteolytic activity	**−**	**this study**

1 Cells synchronized before treatment.

2 Strain-dependent.

**Figure 4:**
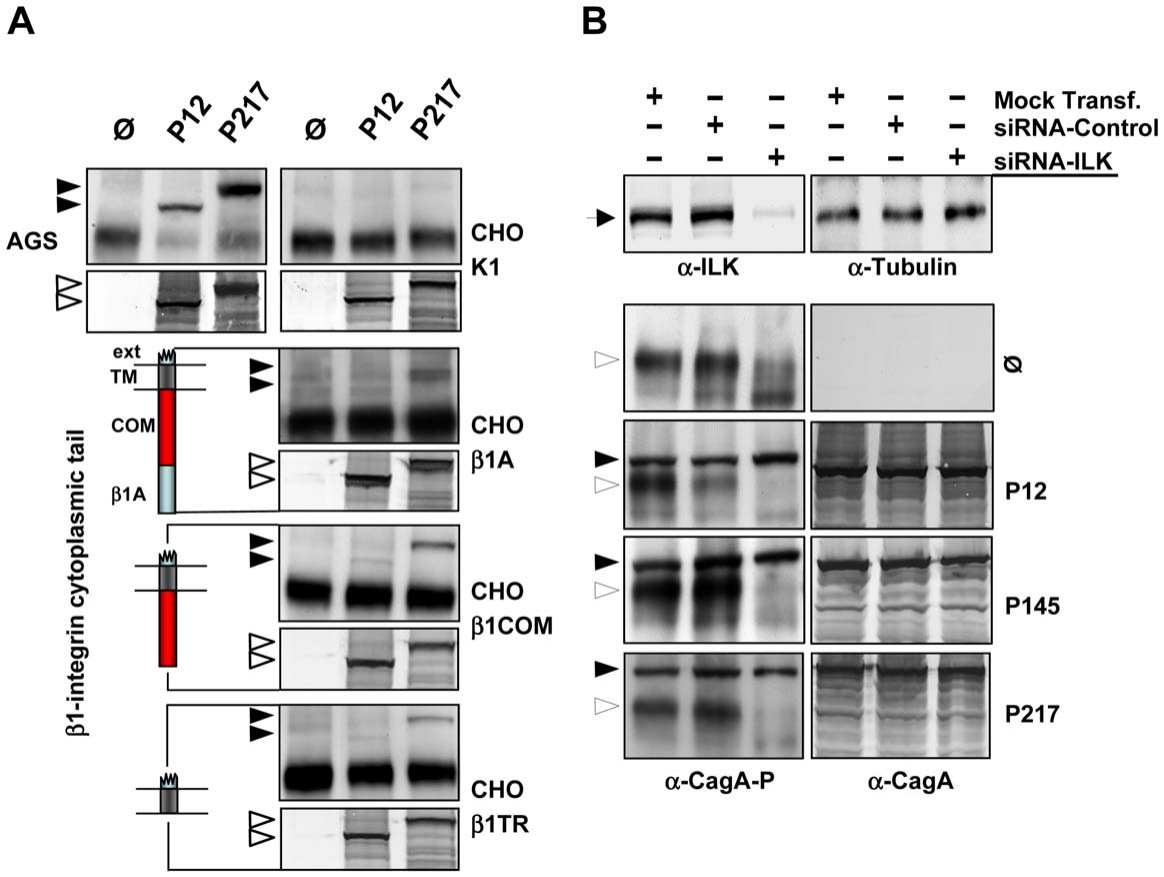
β1 Integrin signalling is dispensable for CagA translocation. (A) CagA translocation assay using *Hp* strains P12 and P217 and CHO cells stably expressing either no (CHO K1), the β1A (complete gene), the β1COM (only common region of cytoplasmic tail) or the β1TR (no cytoplasmic tail) version of the human β1 integrin gene. For a full description of the truncated β1 gene constructs please refer to [25]. Filled arrowheads depict CagA-P, open arrowheads CagA. ext, integrin external region, TM, transmembrane region. (B) Lysates of AGS epithelial integrin linked kinase (ILK) knockdown cells (siRNA-ILK) and cells transfected with GC-matched oligonucleotides (siRNA-Control) or lipofectamine-transfection (Mock-Transfection) were immunoblotted with α-ILK (Sigma) and anti-tubulin antibodies (loading control). The level of ILK knockdown was determined to be 86% by densitometry. AGS wt, ILK knockdown or control cells were infected with *Hp* strains, as indicated. Cell lysates were immunoblotted with α-phospho-tyrosine (PY99) or α-CagA antibodies (AK257). Bands representing CagA-P or CagA are marked by filled arrowheads, open arrowheads depict tyrosine-phosphorylated host cell proteins.

To clarify whether β1 integrin-mediated signalling might be necessary for CagA translocation, we used CHO cells stably transfected with either a full-length human β1A gene, a deletion comprising the complete cytoplasmic tail (β1TR) or constructs containing only the transmembrane and the common region of the β1A tail (β1COM) [25]. Surface expression of β1A integrin was verified by flow cytometry (data not shown). With exception of the CHO vector control, all cell lines were competent for CagA translocation by *Hp* P217 and with lower efficiency in the P12 strain (Figure 4A). The generally low efficiency of CagA translocation into β1 reconstituted CHO cells might be due to a combination of human β1 integrin with the endogenous hamster α integrin chains. Nevertheless, these data suggest that the cytoplasmic tail of β1 integrin and therefore outside-in signalling via the integrin β1 chain is not essential for CagA translocation, although these data do not exclude that such a signalling occurs under in vivo conditions.

To further substantiate these findings and to prove whether CagA translocation via the T4SS may occur independently of β1 integrin signalling, we applied an integrin linked kinase (ILK) gene knockdown. ILK binds to the β1 integrin cytoplasmic domain, thereby directly coupling outside-in integrin signalling to a variety of downstream signal transduction pathways [26]. Production of the ILK protein was reduced by ∼80% at 60h after transfection of the ILK siRNA (Figure 4B). Interestingly, knockdown of ILK had no effect on the capacity of CagA translocation by any of the three *Hp* strains used (Figure 4B), providing strong evidence that CagA translocation solely depends on the extracellular part of β1 integrin and its clustering in lipid rafts, but does not necessarily need the β1 integrin/ILK downstream signalling pathway.

### Interference with CagA Translocation

β1 integrin heterodimers binding to ligands, such as fibronectin, collagen or laminin, involves the α chain and the β chain [27]. Integrin head domains are able to adopt two alternative conformations, termed open (high affinity) and closed (low affinity), which are modulated via binding of metal ions, such as Ca^2+^ (stabilising closed conformation, deactivating), or Mg^2+^ or Mn^2+^ (stabilising open conformation, activating). [27]. Deactivation of integrins by treatment with Ca^2+^ or EDTA did not interfere, but the intracellular Ca^2+^-chelator BAPTA significantly reduced either translocation or tyrosine-phosphorylation of CagA in AGS cells. In contrast, extracellular activation of integrins (Mn^2+^) significantly enhanced CagA translocation and its tyrosine-phosphorylation (Table 1). Treatment of AGS cells with proteases, such as trypsin or thrombin, which leads to detachment of the cells, but not cleavage of integrin heterodimers, resulted in slightly enhanced, rather than abrogated CagA translocation efficiency (Table 1). This observation might possibly be explained by an indirect activation of β1 integrin through Proteinase-Activated Receptor-2 (PAR-2) [28]. Natural ligands of α5β1 integrin, such as fibronectin, *Yersinia enterocolitica* invasin [29] or RGD peptides, even in high quantities, did not alter CagA translocation efficiency (Table 1).

**Figure 5:**
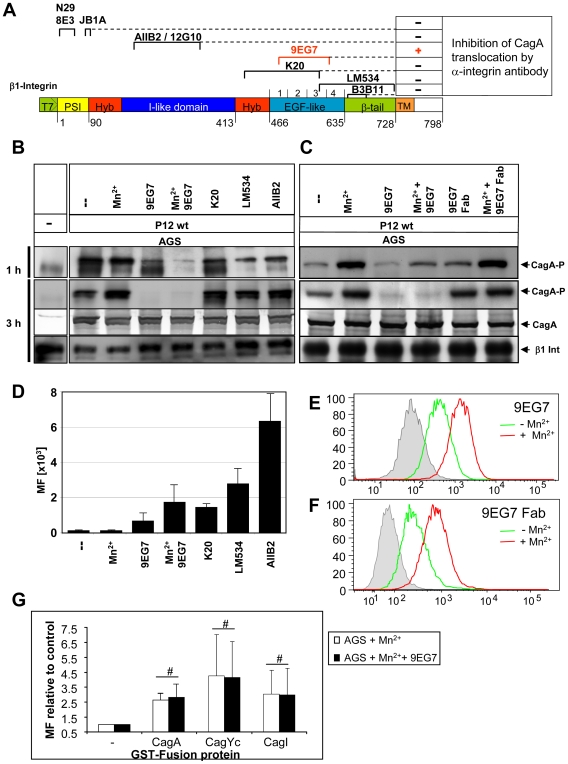
Interference with CagA translocation using β1 integrin-specific monoclonal antibodies. (A) Mapped binding sites of anti-β1 integrin mAbs and their capacity to block CagA translocation are indicated (see also Table 1). (B) Pre-treatment of synchronized AGS epithelial cells by β1-specific mAbs (30µg, 1h or 3h), or (C) Pre-treatment of synchronized AGS epithelial cells by mAb 9EG7 or its Fab fragments generated by papain digestion (15µg, 1h or 3h). After *Hp* infection CagA translocation was determined (CagA-P, tyrosine-phosphorylated CagA, PY99), CagA and β1 Integrin were used as loading controls. (D) Verification of β1 integrin antibody binding to AGS cells, as determined by FACS (MF, mean fluorescence (α-mouse and α-rat AlexaFluor_488_). (E) Quantification of 9EG7 binding to AGS cells with or without Mn^2+^ treatment by flow cytometry. (F) Quantification of 9EG7 Fab fragment binding to AGS cells with or without Mn^2+^ treatment by flow cytometry. (G) Quantification of binding of GST-CagA, GST-CagYc or GST-CagI to AGS cells treated with Mn^2+^ or Mn^2+^ and mAb 9EG7 by FACS analysis, to determine a possible competition in binding of GST fusion protein and 9EG7. # indicates no significant difference.

We next used a set of specific anti-β1 monoclonal antibodies (mAbs), including stimulatory (N29, 8E3, 12G10, 9EG7, B3B11), inhibitory (JB1A, AIIB2), and neutral ones (K20, LM534), to check for interference with the binding of Cag proteins to β1 integrin and thus eventually block CagA translocation (Table 1). Antibodies targeting different domains of β1 integrin were shown to bind to AGS cells (Figure 5A,D). These well-defined mAbs cover essentially all domains and conformations of the β1 integrin chain (Figure 5A), but with the exception of 9EG7, none of them was able to interfere with CagA translocation (Figure 5B,C and Table 1). According to its function, mAb AIIB2 detached AGS cells from the tissue culture plate. This is due to its interaction with the ligand binding domain and its β1 integrin deactivation, but this binding did not block CagA translocation (Figure 5B). Thus, our data show a novel type of interaction of the *cag*-T4SS with β1 integrins, which is independent of the integrin α chain and a typical integrin/ligand interactions, as well as RGD-motifs in any of the Cag proteins.

### Locking Integrin in its High Affinity Conformation Blocks CagA Translocation

Several studies have indicated that a close apposition of the α and β subunits in the membrane-proximal region and the so-called bent structure of the heterodimer are characteristic for the low affinity state of integrins [30],[31]. In contrast, the extended conformation, characterized by separated legs (comprising the β1 I-EGF1-4/β-tail and the α chain Calf-1/Calf-2 domains), represents the high affinity state [15]. Certain allosteric β1 integrin antibodies are able to modulate integrin activity rather specifically by stabilizing a distinct affinity state of the integrin [32]. 9EG7 is a β1-specific mAb with a binding epitope in the I-EGF2-4 region, which is strongly exposed upon manganese treatment or ligand binding (Figure 5E) [33]. The mAb 9EG7 stabilizes and probably fixes conformational changes in the integrin heterodimer, which means that 9EG7 binding of activated integrin will no longer allow its inactivation/bending.

Interestingly, mAb 9EG7 completely blocked CagA translocation in AGS cells, whereas Mn^2+^ alone enhanced, rather than reduced CagA translocation (Figure 5B). A papain-generated Fab fragment of mAb 9EG7 binds in a Mn^2+^-dependent way to the integrin receptor (Figure 5F), but is unable to block CagA translocation (Figure 5C). A direct competition for binding of 9EG7 and GST-Cag proteins to the same integrin epitope was excluded, as measured by FACS analysis (Figure 5G).

**Figure 6:**
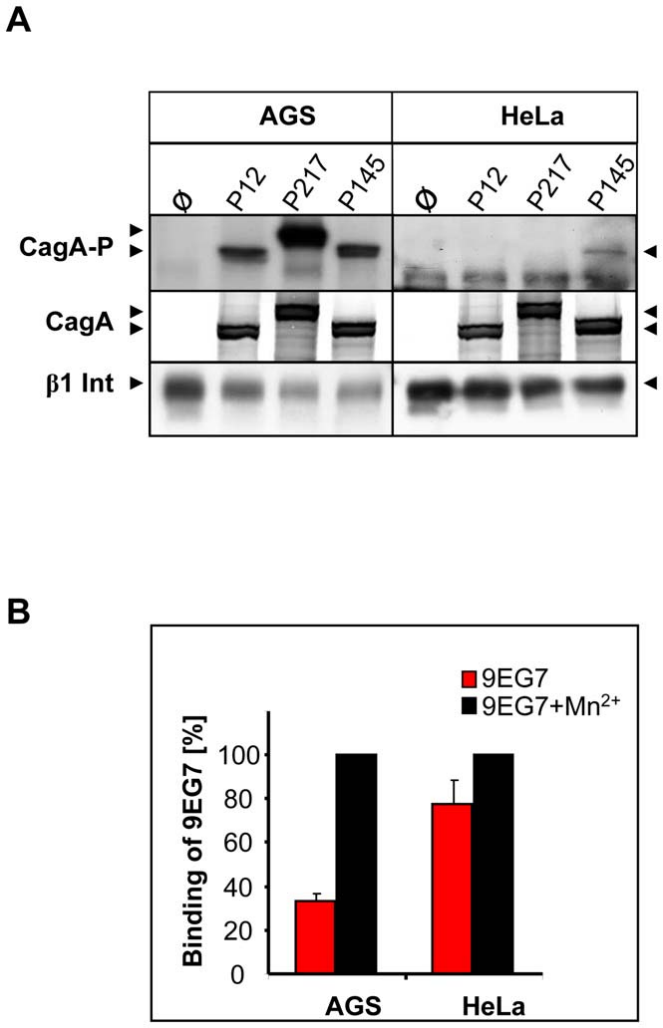
HeLa cells are inefficient for CagA translocation and produce constitutively active β1 integrin. (A) Human gastric AGS and HeLa cells were infected with *Hp* strains P12 P217 and P145 and CagA translocation was determined by immunoblotting with antibody PY99 (CagA-P). Equal loading of bacteria and cells was determined by CagA (AK257) and β1 integrin detection (LM534). (B) Quantification of binding of mAb 9EG7 to AGS or HeLa cells with or without Mn^2+^ treatment by flow cytometry.

The important function of the integrin activation state was supported by the human cervix cell line HeLa. This cell line produced normal levels of β1 integrin on the cell surface, as determined by flow cytometry (data not shown), but was only very inefficiently, or not at all able to act as host cell for CagA translocation by certain *Hp* strains (Figure 6A). An *in vitro* phosphorylation assay ruled out a possible defect in CagA tyrosine phosphorylation (data not shown). The binding capacity of mAb 9EG7 revealed a 20% difference between non-activated and activated (Mn^2+^) state of the cells, whereas for AGS cells the difference was approx. 65%. These data suggest that, due to an unknown mechanism, HeLa cells apparently produce constantly activated β1 integrin, which might be locked in the active state unable to switch back to the closed, inactive conformation, similar to the situation obtained by 9EG7 binding. This could explain the limited ability of *Hp* to translocate CagA.

## Discussion

The *cag*-Type IV Secretion System (*cag*-T4SS) of *Hp* constitutes one of the most important virulence factors of this gastric bacterial pathogen. The mechanism by which *Hp* translocates CagA into host epithelial cells is still not well understood. An important finding was that the *cag*-T4SS apparently does not inject its effector protein CagA randomly into target cells, but uses the α5β1 integrin as a cellular receptor for the pilus-associated adhesin CagL [14]. CagL is the only *cag*-PAI encoded protein carrying an RGD sequence, which is present in certain extracellular matrix proteins and known as a typical integrin/ligand interaction motif [34].

In the present study, we describe the mammalian β1 integrin in different combinations with integrin α chains as a receptor for the *Hp* T4SS. Convincing data for a functional role of β1 integrin were obtained by the promyelocytic HL60 cell line. Non-differentiated promyelocytic HL60 cells, producing high levels of β1 integrin on their cell surface, but not differentiated dHL60 cells, with low levels of surface-associated β1, translocated CagA very efficiently (Figure 1A,C). These data were substantiated by using integrin-deficient murine fibroblast (GD25) or epithelial (GE11) cells, which were completely resistant to CagA translocation by *Hp*, but could be functionally restored upon re-expression of the β1 integrin (GD25β, GE11β) (Figure 1B).

**Figure 7:**
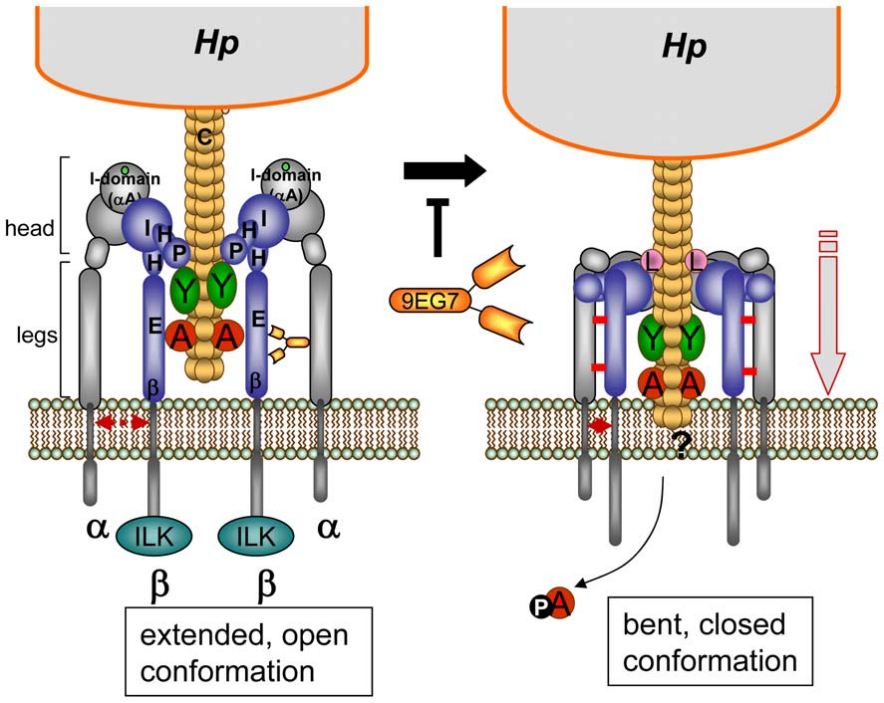
Proposed working model for β1 integrin acting as a receptor of the *cag*-T4SS pilus for translocation of CagA protein. *Hp* T4SS pili (consisting of the pilus subunit CagC) are decorated by proteins CagA, CagY, CagL and probably CagI. The pilus associated proteins contact β1 integrin in the open extended conformation of the head, leading to the clustering of the integrin heterodimers. These binding events may induce a change at the legs of the integrin α/β chains, moving both legs together, which results in a closing and downward movement of the integrin head domains (bent, closed conformation) [39], which seems to be essential for CagA delivery. The mAb 9EG7 binds to the EGF-like domains of the β1 legs and stabilizes the open conformation by preventing a close interaction between the α and β integrin legs (disulfide bridges, indicated by red bars), which is suggested to prevent the downward movement and therefore a possible membrane insertion of the T4SS pilus and thus CagA delivery. α, alpha integrin subunit; β, beta integrin subunit; ILK, integrin-linked kinase; A, CagA; Y, CagY; L, CagL protein; E, EGF-like1-4; β, β-tail domain; H, Hybrid; I, I-like; P, PSI domain, 9EG7, mAb 9EG7.

Here, we performed a systematic YTH screen to identify all proteins of the *cag*-PAI interacting with the β1 integrin receptor. We identified the translocated effector protein CagA, the C-terminal domain of the secretion apparatus component CagY and CagI as binding partners of the integrin receptor. Biochemical evidence for a receptor function of α1β1 (a collagen and laminin receptor) or α5β1 integrin (a fibronectin receptor) was obtained by (i) pulldown experiments using *Hp* lysates (Figure 2B), (ii) direct binding of the corresponding GST-Cag fusion proteins to β1 integrin as determined by FACS analysis (Figure 2C), or (iii) by surface plasmon resonance measurements (Figure 3C,D). CagA binds β1 integrin with a significantly lower K_D_ value as α5β1 integrin binds in a RGD dependent way to fibronectin, its natural ligand. CagA affinity for αVβ3 is significantly lower (approx. 100-fold) as compared to α5β1, demonstrating the high specificity of CagA for the β1 heterodimer. CagA also binds with significantly higher affinity as postulated for CagL to α5β1 integrin. CagA carries a C-terminal translocation signal, but also the N-terminus is essential [19]. So far, the role of the N-terminal portion of CagA had remained elusive, but this specific binding to β1 could explain its important role in the translocation process. The exceptionally high affinity of CagA for the integrin receptor might compensate for the relative low abundance of CagA at the tip of the *cag*-T4SS pilus and suggests an important function for the surface-associated CagA. The integrin binding might have a structural role in triggering integrin rearrangements, whereas only the cytoplasmic (non-pilus-associated) CagA might act as the translocated effector molecule when a translocation-competent configuration has been established (see model Figure 7).

Whereas our data support the essential role of β1 integrin for the process of CagA translocation, the RGD-dependent binding of CagL to the integrin receptor could not be verified. Using *Hp* extracts, we were able to show here a direct interaction of native (non-recombinant) CagA or CagY with integrin heterodimers, however we could not confirm an interaction of native CagL with the α5β1 integrin. It is possible that in the native pilus-associated CagL the RGD motif is buried within the protein and not accessible to interaction. In recombinant overexpression systems, this motif could be exposed, due to partially incorrect folding. Our genetic complementation data support this theory. We are able to successfully rescue CagA translocation with the complementation of CagL mutants, independently of the RGD status of the protein. Possible failures in complementation are known for *Hp* due to frequent secondary mutations, often in the *cag*-PAI [35]. In support of these genetic data we also showed that binding of GST-CagL protein to β1 integrin is very low, as compared to CagA, CagI or CagYc. More important, the binding of purified GST-CagL to β1 integrin was completely independent from its RGD motif. Thus, we show on the functional as well as on the binding level that the RGD motif of CagL is not essential for the protein function.

To further study the type of interaction between the β1 integrin heterodimer and the Cag proteins, we used typical β1 integrin ligands, such as RGD peptide, fibronectin or *Yersinia* invasin protein, to possibly interfere with CagA translocation (Table 1). None of these known ligands was able to block CagA translocation, indicating that the Cag proteins use different sites on the integrin for interaction. Kwok et al showed that *Escherichia coli* strain HB101 that expresses Yersinia invasin inhibited CagA phosphorylation in AGS cells. It might be possible that *E. coli* expressing invasin on the surface could sterically inhibit *H. pylori* to bind and translocate CagA, just by blocking the access to the integrins.

Interaction of integrins with its ligands results in integrin clustering. Inhibition of integrin clustering into lipid rafts using methyl-β-cyclodextrin or calpeptin strongly reduced CagA translocation, indicating that *Hp*-mediated clustering of β1 integrin heterodimers on the cell surface might be essential for this process. To determine whether integrin signalling might play a role for CagA translocation, signalling-deficient, truncated versions of β1 integrin receptor were used. Unexpectedly, neither the β1 integrin cytoplasmic tail, nor signalling via the integrin linked kinase was necessary for CagA translocation, indicating that only the extracellular domains of the β1 integrin is important. CHO cells derived from hamster generally showed a lower CagA translocation efficiency as compared to human AGS cells (Figure 4A). We assume that human/hamster integrin heterodimers, generated upon transfection are the reason, an effect also seen for murine GE11 cells (human/mouse integrin heterodimers) (Figure 1B). *H. pylori* P217 shows a very strong CagA phosphorylation in AGS cells due to its high number of EPIYA motifs (8 motifs as compared to 4 in P12), resulting in more efficient tyrosine phosphorylation as P12 in CHO cells.

To obtain insight which domains of the extracellular part of the integrin receptor are important, a set of defined monoclonal antibodies against various β1 integrin domains were applied (Figure 5A, Table 1). None of these antibodies, even those blocking the integrin ligand interaction (AIIB2, 12G10), were able to block CagA translocation, except mAb 9EG7. This is in contrast to many viruses, which use integrins as receptors or co-receptors for entry into different host cells [36]–[38]. Most viruses known to use integrins as entry receptors have been shown to do so by extracellular matrix (ECM) protein mimicry, which means that viral proteins contain an RGD or any other conserved integrin recognition motif. Thus, specific antibodies, which block integrin ligand interaction, usually abrogate virus infection *in vitro* [36]–[38]. Taken together our data suggest that the *cag*-T4SS uses the extracellular portion of integrin to mediate entry of the effector protein into the cell by a different mechanism, probably independent from ECM protein mimicry and the usual integrin ligand interaction.

What is the difference in the effect of mAb 9EG7 in comparison to all the other β1 specific mAbs used in this study? 9EG7 binds an epitope in the β1 integrin which is close to the fulcrum at the genu and is buried in the inactive (bent) state of the integrin receptor. Mn^2+^ treatment or ligand binding opens the integrin into the extended conformation and the epitope is free for antibody binding. When 9EG7 is bound, the integrin cannot move back into the bent conformation, probably due to sterical problems with the bulky Fc part of the antibody or its ability to crosslink integrin chains. In addition, we cannot exclude the possibility that 9EG7 might prevent the interaction of the β1 integrin subunit with a co-receptor necessary for the translocation process, although there is no evidence for a co-receptor being involved. The 9EG7 Fab fragment still needs activation of the integrin for binding (Figure 5E,F). This indicates that its binding is unchanged, but the lack of the Fc chain will not cause the effects presumed for the complete mAb, due to the smaller size of the Fab fragment and its inability to crosslink. Our data lead us to propose a model whereby the rearrangement of the integrin from the extended, open conformation, binding the T4SS components, to a bent conformation (bent closed) is an essential step in the process of CagA translocation (see Figure 7 for a model). We propose that this mechanics of the integrin, which is associated with a closer approximation of the integrin head to the cellular membrane [27], brings the pilus closer to the cellular membrane. When this conformational change is inhibited, CagA translocation cannot occur.

## Materials and Methods

### Bacterial strains, cell lines and culture conditions

#### *Hp* Strains

*Hp* strains P12, P145 and P217 and the isogenic knockout mutants P12Δc*ag*A, P12Δ*cagE*, P12Δ*cag*Y as well as P12ΔPAI and P217ΔPAI, lacking the entire *cag* pathogenicity island, have been constructed as previously described for the corresponding mutant strains in *Hp* 26695 [11]. *Hp* strains were grown on GC agar plates (Difco) as described previously [11].

#### Eukaryotic cell lines

Eleven different eukaryotic cell lines were analyzed for their capacity to tyrosine-phosphorylate *Hp* translocated CagA and IL-8 induction (Table S1). Cells were grown in media as indicated and subcultured every 2 to 3 days.

### Antibodies and reagents

Antibodies against phosphotyrosine were obtained from Santa Cruz (PY99) or Upstate (4G10), polyclonal horseradish peroxidase (HRP) and alkaline phosphatase-conjugated anti-mouse IgG, anti-rat IgG and anti-rabbit IgG antisera, HRP-conjugated streptavidin, Heptakis(2,6-di-O-methyl)-β-cyclodextrin (Heptakis), fibronectin from human plasma, RGD (Gly-Arg-Gly-Asp-Ser-Pro-Lys) and RAD (Gly-Arg-Ala-Asp-Ser-Pro-Lys) peptides, protease inhibitors PMSF, Leupeptin and Pepstatin were obtained from Sigma. Purified human α1β1 (from smooth muscle), α5β1 (from placenta) and αVβ3 (from placenta) were purchased from Clontech (Millipore). Integrin α5β1 and monoclonal anti-β1 integrin Clone LM534 were purchased from Chemicon International. CD29 FITC and CD18 PE were purchased from BD Biosciences, and anti-β1 integrin antibody (Clone 4B7) was from Calbiochem. Rat anti-human β1 integrin inactivating antibody AIIB2 was extracted from hybridoma cells supernatant. For antibodies and their sources see Table 1. To detect CagL, a rabbit antibody against a purified CagL fusion protein was used [18].

### Yeast two hybrid assay

The Invitrogen system consisting of the entry vector pDONR207 and the destination vectors pDEST-GADT7 (prey vector) and pDEST-GBKT7 (bait vector) were used. Yeast two-hybrid bait and prey libraries were generated comprising the external β1 integrin gene sequence and the *cag*-PAI genes. For the *cag*-PAI genes, 22 full-length open reading frames (excluding N-terminal signal sequences), and 10 partial open reading frames were amplified from chromosomal DNA of strain 26695 by nested PCR, and cloned in the bait and prey vectors exactly as described [18]. Bait and prey plasmids were transformed into the haploid *Saccharomyces cerevisiae* strains Y187 and AH109. Diploid yeast cells were selected after mating and selection on SD medium lacking tryptophan (Trp^−^) and leucine (Leu^−^), thus generating all possible combinations of bait and prey plasmids. After growth on SD-Trp^−^Leu^−^ medium, yeast colonies were transferred to SD-Trp^−^Leu^−^His^−^ medium in order to select for interactions. Growth after 3 to 6 days indicated bait-prey interactions. Additionally, the stringency of this screen was enhanced by selection on SD-Trp^−^Leu^−^His^−^ medium containing the competitive inhibitor 3-aminotriazole (5 mM).

### Phosphorylation assays

Cells were infected with *Hp* at 70–90% confluency with a multiplicity of infection (MOI) of 60. For synchronization experiments, cells were detached with PBS/2 mM EDTA, seeded and after 24 hours synchronized overnight in serum free media. Before infection, RPMI (GIBCO) complete media (CM) containing 10% Fetal Calf Serum (GIBCO) was added to cells, counting this point as time 0. To test different inhibitors, 1, 2 and 4h infections were performed after 60 min from addition of CM. After infection, supernatants from 2 & 4h experiments were collected, cells were harvested in PBS with protease inhibitors (pepstatin 1µM, leupeptin 1µM, PMSF 1mM) and phosphatase inhibitor sodium vanadate (1mM). Harvested cells were centrifuged at 500×g for 10 min at 4°C and pellets lysed in RIPA buffer with protease and phosphatase inhibitors and DNase I for later SDS-PAGE and immunoblot analysis under non-reducing conditions.

### Pull-down assays and immunoblotting

SiMAG magnetic beads (Chemicell) were coated with 50 µg α5β1 integrin/10 mg beads following the manufacturer's instructions. Beads were saturated with 1 M Tris-HCl (pH 7.5). 1 ml *Hp* (OD_550_ of 2) in PBS with protease inhibitors was treated with lysozyme (10 mg/ml, 4 mM EDTA) for 30 min at RT, DNase I was added (1 µg/ml) and bacteria were lysed by ultrasonication on ice. Soluble proteins (Soluble I) and membranes were separated by ultracentrifugation. Membranes were resuspended in 1 ml HSL (High Salt Lysis, 25mM Tris-HCl, pH 7.4, 0.05% Triton-X100, 4 mM MgCl_2_, 3 mM MnCl_2_, 150 mM NaCl) buffer with protease inhibitors, sonicated and centrifuged at 4°C, 13.000 rpm for 1min to collect the soluble fraction (Soluble II). Soluble II was used for protein pulldown. After pulldown, 3µl beads were incubated at 4°C for 1 h, washed 3 times with HSL 400 (HSL with 400 mM NaCl) buffer, boiled and used for SDS-PAGE (non-reducing conditions). Proteins were transferred to PVDF membranes, and blotted with the antisera indicated. Blots were routinely stripped and reprobed with the indicated antisera (α-actin or α-β1 integrin antibodies) as loading controls. Blots shown are representative of three independent experiments.

### Live cell imaging

For co-localization experiments the integrin β1-specific monoclonal antibody 4B7 was labelled with AlexaFluor_568_ according to the manufacturer's instructions (10 mol Alexa Fluor568/mol antibody). AGS cells were grown in 35 mm glass bottom dishes (MatTek) to 60–70% confluency. Cells were washed once with PBS (without Ca^2+^ and Mg^2+^) and infected with GFP-expressing P12 wt or P12ΔPAI grown on serum-free media at an MOI of 60. 2 µg/ml Alexa Fluor 568-labelled antibody against β1 integrin was added. Infection was performed for 7 min. at 37°C and PBS was exchanged before microscopy studies. For quantification of co-localization, assays were recorded over a time range of 50s and picture sequences were analyzed for co-localization events of single bacteria and integrin β1 clusters. Percent co-localization was calculated from ratio of bacteria co-localizing with integrin to total adherent bacteria.

Imaging was done using an UltraView LCI spinning disc confocal system (PerkinElmer) fitted on a Nikon Eclipse TE300 microscope equipped with a temperature- and CO_2_-controllable environment chamber. Images were taken with the black/white ORCA ER Camera (Hamamatsu). Pictures were taken and edited using LCI UltraView software. For immunofluorescence assays, an Olympus BX 64 microscope and Cell ∧P software were used.

### Purification of CagA 100 kDa fragment

CagA gene was cloned into a vector expressing an TEV cleavable His-tag fusion CagA (pHAR3011–CagA) as described in [22]. Briefly, the protein was expressed in BL21 cells induced o/n at 20°C with 1mM isopropyl-β-D-galactopyranoside (IPTG). Harvested cells were lysed by sonication in buffer A (10 mM Na Phosphate pH7.5, 5mM immidazole, 500 mM NaCl, 10% glycerol) containing DNaseI, lysozyme, one mini complete EDTA-free protease inhibitor cocktail tablet (Roche). After centrifugation, the supernatant was loaded onto a His-trap column (GE Healthcare) and eluted with a linear gradient of immidazole (5 to 500 mM) in buffer A. Two major N-terminal fragments (29 kDa and 100kDa, assessed by the His-tag presence) were eluted together with minor degradation products. Fractions were concentrated and the buffer was exchanged to 10mM Tris pH 7.5, 150mM NaCl. The two fragments were separated by gel filtration using Superdex S75 10/300 GL column (GE Healthcare). Fractions containing ∼95% pure 100 kDa fragment (residue 1 to approximately 885) were pooled, concentrated and reloaded on the same column to ascertain stability. The protein was finally concentrated to 1.8 mg/ml.

### Surface plasmon resonance

Using a BiacoreX unit, the purified 100 kDa N-terminal fragment of CagA was attached to a CM5 chip (BiaCore) using the standard amine coupling procedure. The flow-cell 1 was treated similarly without coupling of a protein and was used as a reference. Integrin binding to the reference was negligible. For the evaluation of the interaction of proteins, a Tris buffer was used containing 24 mM Tris-HCl pH 7.5, 137 mM NaCl and 2.4 mM KCl. Injection of the integrin proteins was for 1 min using a flow of 60 µl/min. Subsequently, dissociation was evaluated for 200 sec. Regeneration of the chip took place between each measurement using a solution of Tris 20 mM pH 7.4, 150 mM NaCl, 1 mM MgCl_2_, 1 mM CaCl_2_ and 0.0125% Triton, resulting in a stable baseline and retaining activity. BiaEvaluation software (version 4.1) was used for the evaluation of the dissociation constant using a 1∶1 langmuir model of binding.

### Generation of 9EG7 Fab fragments

1 mg of 9EG7 mAb (rat) (BD Biosciences) was digested using beads coupled to Papain (Pierce) following the manufacturers' instructions. Fab fragments were detected by western blotting using a rabbit anti rat Fab (Rockland Immunochemicals). Binding capacity of the Fab fragments to AGS cells was evaluated by flow cytometry. Secondary antibodies anti-rat Alexa_488_ and anti-rabbit Alexa_488_ were from Molecular Probes.

### Statistical analysis

Data are presented as mean+/−SEM. Differences between groups were assessed by the paired, two-tailed Student's *t*-test, or by the Mann-Whitney *U* test for unpaired groups depending on the data set of concern (see figure legends).

## Supporting Information

Protocol S1 Supporting methods file
(0.04 MB DOC)

Table S1 Cell lines used in this study and their growth conditions
(0.04 MB DOC)

Table S2 Oligonucleotides used for the generation of GST fusion proteins
(0.05 MB DOC)

Figure S1 Protein-protein interactions among *H. pylori cag*-PAI proteins and β1 integrin. Yeast cells co-transformed with plasmids expressing β1 integrin and either HP0527a (CagYa), HP0527b (CagYb), HP0527c (CagYc), HP0540 (CagI), HP0547a (CagAa) or HP0547b (CagAb), growing on minimal SD medium lacking tryptophan and leucine (SD-Trp-Leu) or on selective minimal SD medium lacking tryptophan, leucine, and histidine (SD-Trp-Leu-His). Negative and positive controls were used as described.
(0.77 MB TIF)

Figure S2 Integrin binding assays. (A) General procedure to generate *Hp* extract for pulldown assay with β1 integrin beads. Total lysate depicts the presence of CagA and CagY in P12 wt strain. (B) Quantification of binding of purified GST-Cagβ, GST-CagG, GST-CagZ (negative controls for integrin binding) or GST-Inv397 (positive control) (30mg/1×10^6^ cells) to integrin-deficient GE11 versus integrin-proficient GE11β cells by flow cytometry (n = 4). (C) Quantification of binding of purified GST-CagL or GST-CagL(RAD) (30mg/1×10^6^ cells) to integrin-deficient GE11 versus integrin-proficient GE11β cells by flow cytometry (n = 6). Binding was analysed by anti-GST antibody (fold increase in binding versus GST, the values for binding of GST alone has been set to 1). (*, p<0.05; **, p<0.01; ***, p<0.001; students T-test). MF, mean fluorescence.
(0.63 MB TIF)

Figure S3 High resolution FESEM micro-graphs of AGS cells infected with *H. pylori*. (A–F) CagA labelling is repeatedly found on the tip region of the secretion pilus using immunogold labelled antibody AK273 (arrows) (G–I) P12ΔPAI strain showing only few gold-particles on the AGS cell surface (arrows in G and H); in control experiments with *H. pylori*-infected AGS cells incubated with the gold marker alone no labelling in the tip region of the secretion apparatus is detectable. The background labelling was comparable to the ΔPAI strain (arrows in I). (K–L) Localization of CagY protein on the OM and the *cag*-T4SS pilus of Hp 26695 wt, but not 26695ΔPAI using immunogold-labelled antiserum AK273 by FESEM. Bars represent 200 nm (A–F,K), 1 µm (G), 500 nm (H,I,L).
(6.78 MB TIF)

Figure S4 Pharmacological Iinhibitors interfering with CagA translocation in AGS epithelial cells. CagA translocation into AGS cells is inhibited by calpeptin (CP), an inhibitor of the Ca2^+^-dependent protease calpain. CP, calpain. Filled arrowheads mark CagAP-tyr, open arrowheads mark CagA bands.
(0.79 MB TIF)
