# Supplementary material for: Helicobacter pylori Type IV Secretion Apparatus Exploits β1 Integrin in a Novel RGD-Independent Manner
Source: PLoS Pathog. 2009 Dec 4;5(12):e1000684. doi: 10.1371/journal.ppat.1000684 (PMC2779590; doi:10.1371/journal.ppat.1000684)
Supplement: Protocol S1 — Supporting methods file (0.04 MB DOC) [file ppat.1000684.s001.doc]

**Supporting Methods**

***Helicobacter pylori* Type IV secretion apparatus exploits β1 integrin in a novel RGD-independent manner**

Luisa F. Jiménez-Soto^1^, Xaver Sewald^1^, Stefan Kutter^1^, Claudia Ertl^1^, Evelyn Weiss^1^, Ulrike Kapp^2^, Manfred Rohde^3^, Torsten Pirch^4^, Kirsten Jung^4^, S. Francesco Retta^5^, Laurent Terradot^2^, Wolfgang Fischer^1^ and Rainer Haas^1^*

^1^Max von Pettenkofer-Institute for Hygiene and Medical Microbiology, Ludwig-Maximilians-Universität, D-80336 München, Germany

^2^Macromolecular Crystallography Group, European Synchrotron Radiation Facility, F-38043 Grenoble Cedex, France

^3^Helmholtz Center for Infection Research, Department of Microbial Pathogenesis, D-38124 Braunschweig, Germany

^4^Munich Center of integrated Protein Science, CiPSM, at the Department of Biology, Microbiology, of the Ludwig-Maximilians-Universität München, D-82152 Planegg-Martinsried

^5^Molecular Biotechnology Centre, Department of Genetic, Biology and Biochemistry, 10126 Torino, Italy

## *To whom correspondence should be addressed. E-mail: [haas@mvp.uni-muenchen.de](mailto:haas@mvp.uni-muenchen.de)

**This PDF file includes:**

Supporting Experimental Procedures

Supporting References

**Supporting Materials and Methods**

Cell Transfection and Integrin Expression by Flow Cytometry

Low passage cells with 20-30% confluency in 6 wells were transfected with 50nM siRNA using Lipofectamine by the protocol described by the manufacturer. As transfection control a fluorescent siRNA was used, giving 90 – 98% transfection efficiency. After 36h cells were splitted to infection wells. A 2h infection was done after 50-60 hours of the transfection. For the analysis of expression of β1 integrin on the surface of the cell, cells were synchronized overnight in serum free RPMI media when they had reached 70 - 80% confluency. Media containing serum was added (time point 0) and fixation was done using the previously described in flagranti method for 1 h followed by an overnight blocking with 10% FCS in PBS solution. Primary antibody AIIB2 was added for 1 h at 4°C, and secondary anti-rat Alexa488 was added 1:2500 concentrated for 1h at 4°C. Cells were washed and scrapped gently from wells, collected in PBS and read in a FACS Excalibur. Evaluation of FACS data was done using the WinMDI freeware Version 2.8 statistics.

Immuno Field Emission Scanning Electron Microscopy (FESEM)

AGS cells, grown on cover slips, were infected with *Hp* P12 for different time points. Samples were fixed with 3.7% paraformaldehyde in PBS on ice for 1h and left in the fixation solution. Before labelling with antibodies samples were washed three times with cacodylate buffer (0.1 M cacodylate, 0.01 M CaCl_2_, 0.01 M MgCl_2_, 0.09 M sucrose, pH 6.9) containing 10 mM glycine at room temperature. Samples were incubated with protein G-purified anti-CagA antibodies (100 µg/ml IgG protein) diluted in PBS for 2 h at 30°C and washed several times with PBS. Bound antibodies were made visible with 15 nm protein A/G gold-particles for 30 min at 30°C. After washing with PBS samples were fixed with 2% glutaraldehyde for 15 min at room temperature, washed with TE-buffer (20 mM TRIS, 1 mM EDTA, pH 6.9) and dehydrated in a graded series of acetone (10, 30, 50, 70, 90 and 100%), critical-point dried with CO_2_ (Bal-Tec CPD030, Liechtenstein) and coated with a thin carbon film (Bal-Tec SCD500, Liechtenstein). Samples were examined in a Zeiss field emission scanning electron microscope DSM982 Gemini at an acceleration voltage of 5 kV using the Everhart-Thornley and inlens SE-detectors in a 50:50 ratio. Images were recorded onto MO-disks and processed for contrast and brightness applying Adobe Photoshop CS2.

Bacterial Strains and Culture Conditions

*Hp* strains were grown on GC agar plates (Difco) supplemented with vitamin mix (1%), horse serum (8%), vancomycin (10 mg l^−1^), trimethoprim (5 mg l^−1^) and nystatin (1 mg l^−1^) (serum plates), and incubated for 16–60 h in a microaerobic atmosphere (85% N_2_, 10% CO_2_, 5% O_2_) at 37°C. *E. coli* strains Top10 (Invitrogen) and DH5α (BRL) were grown on Luria–Bertani (LB) agar plates or in LB liquid medium supplemented with ampicillin (100 mg l^−1^) or chloramphenicol (30 mg l^−1^), as appropriate.

Additional Reagents

SiMAG Carboxyl magnetic beads were from Chemicell (Berlin, Germany). For immunofluorescence studies, AlexaFluor_488_- and AlexaFluor_555_-conjugated goat anti-rabbit IgG, goat anti-rat IgG and goat anti-mouse IgG antisera and Cy3-conjugated anti-goat IgG antiserum as well the fluorescent reagents AlexaFluor_488_, AlexaFluor_555_ and AlexaFluor_647_ were purchased from Molecular Probes. Calpeptin was purchased from Calbiochem; wortmannin, cytochalasin D, trypsin, thrombin, and fillipin from Sigma. Annealed small interfering RNAs (siRNAs) against ILK sequence 5-AAG GAA GAG CAG GGA CTT CAA-3, had the sense strand sequence r(GGA AGA GCA GGG ACU UCA A)dTdT and antisense r(UUG AAG UCC CUG CUC UUC C)dTdT were purchase from QIAGEN; and siRNA Medium GC Control as well as Lipofectamine^TM^ RNAiMAX Transfection reagent were purchased from Invitrogen.

Protein Staining

Staining of CagA was performed with a protein G-purified polyclonal rabbit antibody AK257 (against C-terminus), labelled with Alexa Fluor647-succinimidyl ester (molecular probes) according to manufacturer´s instructions (10 mol Alexa Fluor647/ mol antibody). For microscopy studies P12-GFP and P12ΔPAI mutant bacteria, respectively, were incubated for 5 min at 37°C with 2µg/ml anti-CagA-Alexa647 in PBS. After removal of unbound antibody, AGS cells were infected with *Hp* for 10 min at 37°C in PBS.

In Vitro Phosphorylation Assay

The phosphorylation of CagA variants using AGS cell lysates was performed as described [1]. Briefly, 2 × 10^7^ AGS cells were lysed in 600 μl of ice-cold NP-40 buffer (20 mM Tris-HCl, pH 7.5, 150 mM NaCl, 50 mM NaF, 1 mM Na3VO4, 1 mM EDTA, 1 mM PMSF, 10 μg ml−1 leupeptin, 10 μg ml−1 pepstatin, 1% Nonidet P-40), and 3 × 10^9^ *Hp* cells were lysed in 1 ml of the same buffer. A mixture of 45 μl of each lysate was incubated together with 5 μl 10× phosphorylation reaction buffer (250 mM Tris-HCl, pH 7.2, 400 μM ATP, 62.5 mM MnCl2, 312.5 mM MgCl2, 625 μM Na3VO4) for 10 min at 30°C. Similar experiments were performed with GD25 or GE11 cells. The lysates were mixed with sample buffer and analysed by immunoblotting.

**Supporting References**

1. Hohlfeld S, Pattis I, Püls, J, Plano GV, Haas R and Fischer W (2006) A C-terminal translocation signal is necessary, but not sufficient for type IV secretion of the *Helicobacter pylori* CagA protein. *Mol. Microbiol.* 59:1624-1637
