# Supplementary material for: Helicobacter pylori Type IV Secretion Apparatus Exploits β1 Integrin in a Novel RGD-Independent Manner
Source: PLoS Pathog. 2009 Dec 4;5(12):e1000684. doi: 10.1371/journal.ppat.1000684 (PMC2779590; doi:10.1371/journal.ppat.1000684)
Supplement: Table S1 — Cell lines used in this study and their growth conditions (0.04 MB DOC) [file ppat.1000684.s002.doc]

| **Table S1: Cell lines used in this study and their growth conditions** | | | |
| --- | --- | --- | --- |
| Cell line | Reference or source^1^ (no.) | Cell type | Growth medium^2^ |
| AGS | ATCC - CRL 1739 | Human gastric adenocarcinoma | RPMI 1640 (Gibco BRL), 10% FBS, 2 mM L-glutamine |
| AIIB2 | Werb et al. 1989 | Rat spleen, hybridoma | DMEM (Gibco BRL), 10% FBS, 2 mM L-glutamine |
| CHO K1 | ATCC - CCL 61 | Chinese hamster ovary | F12 (Gibco), 10% FBS, 2 mM L-glutamine |
| Hela | ATCC - 2 | Human cervix epithelial carcinoma | DMEM (Gibco BRL), 10% FBS, 2 mM L-glutamine |
| J774.A | DSMZ - ACC 170 | Mouse monocytic macrophages | RPMI 1640 (Gibco BRL), 10% heat-inactivated FBS,  2 mM L-glutamine, 10% CO² |
| Jurkat | DSMZ - ACC 282 | Human blood, lymphoblastic leukemia | RPMI 1640 (Gibco BRL), 10% FBS, 2 mM L-glutamine |
| HL-60 | DMSZ – ACC 3 | Human blood, leucemia | RPMI (Biochrom), 10% FBS (Biochrom), 2 mM L-glutamine |
| GD 25 | Wennerberg et al. 1996 | Murine embryonic fibroblast like | DMEM (Gibco BRL), 10% FBS, 2 mM L-glutamine, |
| GD25ß | Wennerberg et al. 1996 | Murine embryonic fibroblast like | DMEM (Gibco BRL), 10% FBS, 2 mM L-glutamine,  10µg/ml Puromycin |
| GE11 | Gimond et al. 1999 | Murine embryonic epithelial | DMEM (Gibco BRL), 10% FBS, 2 mM L-glutamine, |
| GE11ß | Gimond et al. 1999 | Murine embryonic epithelial | DMEM (Gibco BRL), 10% FBS, 2 mM L-glutamine,  0,2 mg/ml Zeocin |
| MDCK | ATCC – CCL34 | Canine kidney epithelial | DMEM (Gibco BRL), 10% FBS, 2 mM L-glutamine |
|  |  |  |  |

1. ATCC, American Type Culture Collection; DMSZ, Deutsche Sammlung von Mikroorganismen und Zellkulturen GmbH
2. DMEM, Dulbecco’s modified Eagle’s medium; F12, F-12 Nutrient Mixture (Ham),
