# Supplementary material for: Helicobacter pylori Type IV Secretion Apparatus Exploits β1 Integrin in a Novel RGD-Independent Manner
Source: PLoS Pathog. 2009 Dec 4;5(12):e1000684. doi: 10.1371/journal.ppat.1000684 (PMC2779590; doi:10.1371/journal.ppat.1000684)
Supplement: Table S2 — Oligonucleotides used for the generation of GST fusion proteins (0.05 MB DOC) [file ppat.1000684.s003.doc]

**Table S2: Oligonucleotides used for the generation of GST fusion proteins**

| **Primer** | **Locus** | **Orientation** | **Restriction site** | **Sequence (5’-3’)** |
| --- | --- | --- | --- | --- |
| **WS293** | **HP524/Cagbeta** | **fwd** | **BamHI** | **GCGGATCCAAATACTTAACTCGGACTAG** |
| **RB31** | **HP524/Cagbeta** | **rev** | **SalI** | **tgggtcgactcacagttcacttgaacc** |
| **SH1** | **HP526/CagZ** | **fwd** | **BamHI** | **CGGGATCCGAACTCGGTTTCAATGAAG** |
| **SH2** | **HP526/CagZ** | **rev** | **XhoI** | **ACCGCTCGAGTTATTCCAAATTTAATTTT** |
| **SH3** | **HP542/CagG** | **fwd** | **BamHI** | **CGGGATCCAAAACGAATTTTTATAAAA** |
| **SH4** | **HP542/CagG** | **rev** | **XhoI** | **TGGCCTCGAGTTAATACCCTAAGATCGGT** |
| **WS435** | **HP539/CagL** | **fwd** | **BamHI** | **CGGGATCCGAAGATATAACAAGCGGCTTAAAG** |
| **JP52** | **HP539/CagL** | **rev** | **XhoI** | **ACCGCTCGAGTCATTTAACAATGATCTT** |
| **WS307** | **HP540/CagI** | **fwd** | **BamHI** | **TAGGATCCCC GGTAATAACG CTTGAACCCG** |
| **WS308** | **HP540/CagI** | **rev** | **XhoI** | **ACCGCTCGAG TCATTTGACA ATAACTTTAG** |
| **LJ38** | **HP527c/CagYc** | **fwd** | **BamHI** | **CGTGGATCCATCATAGCTCTAGATAAACTCATAGGC** |
| **LJ39** | **HP527c/CagYc** | **rev** | **NotI** | **CGTAGCGGCCGCTTAATTGCCACCTTTGGGGCTTGTGGT** |
| **WS315** | **HP547/CagA** | **fwd** | **BamHI** | **CAGGATCCACTAACGAAACCATTAACC** |
| **HK3** | **HP547/CagA** | **rev** | **SalI** | **CGGGATCCGTCGACTTAAGATTTTTGGAAACCAC** |

fwd, forward; rev, reverse
