# Supplementary figures and images for: Helicobacter pylori Type IV Secretion Apparatus Exploits β1 Integrin in a Novel RGD-Independent Manner
Source: PLoS Pathog. 2009 Dec 4;5(12):e1000684. doi: 10.1371/journal.ppat.1000684 (PMC2779590; doi:10.1371/journal.ppat.1000684)

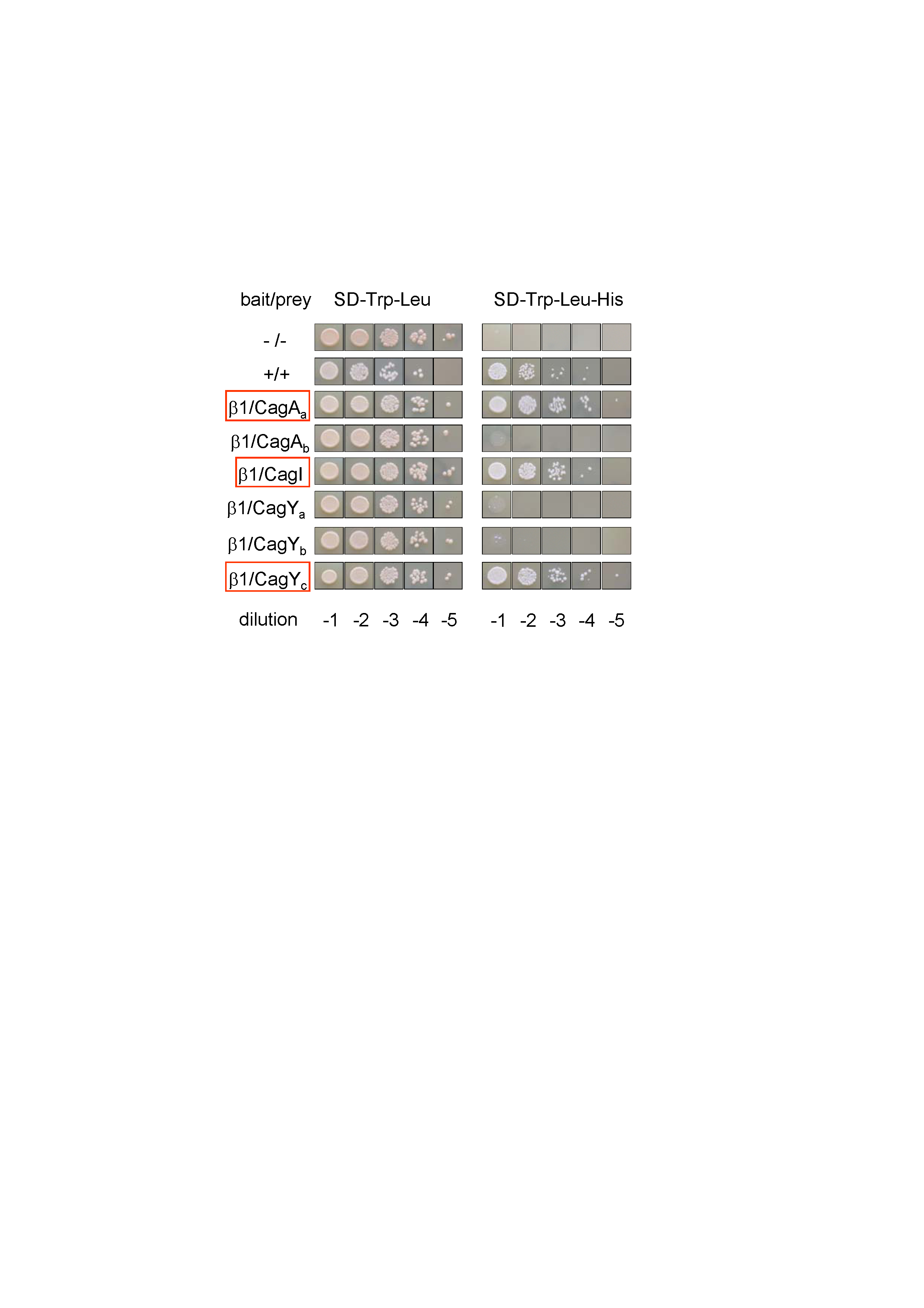

Supplement: Figure S1 — Protein-protein interactions among H. pylori cag-PAI proteins and β1 integrin. Yeast cells co-transformed with plasmids expressing β1 integrin and either HP0527a (CagYa), HP0527b (CagYb), HP0527c (CagYc), HP0540 (CagI), HP0547a (CagAa) or HP0547b (CagAb), growing on minimal SD medium lacking tryptophan and leucine (SD-Trp-Leu) or on selective minimal SD medium lacking tryptophan, leucine, and histidine (SD-Trp-Leu-His). Negative and positive controls were used as described. (0.77 MB TIF) [file ppat.1000684.s004.tif]

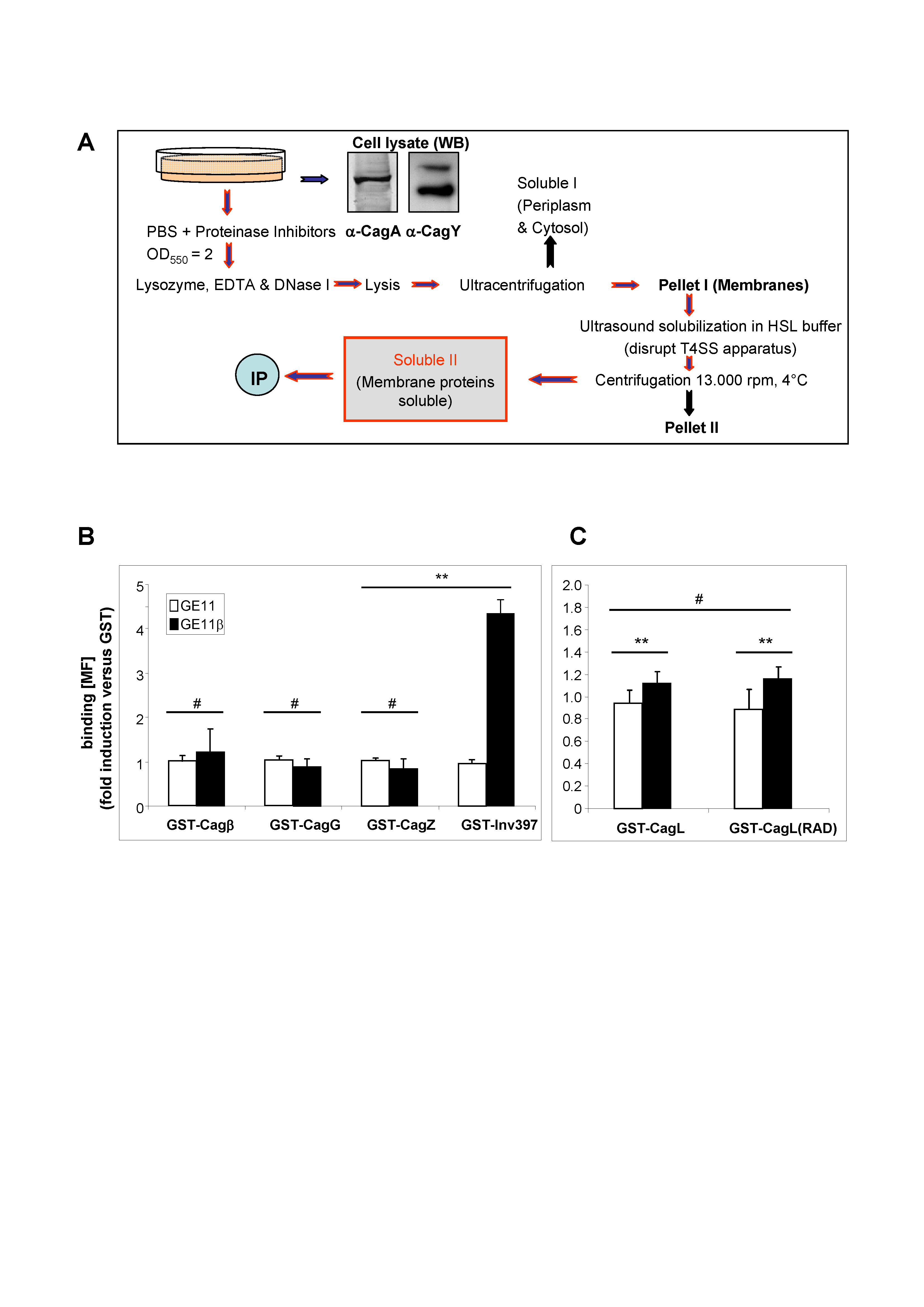

Supplement: Figure S2 — Integrin binding assays. (A) General procedure to generate Hp extract for pulldown assay with β1 integrin beads. Total lysate depicts the presence of CagA and CagY in P12 wt strain. (B) Quantification of binding of purified GST-Cagβ, GST-CagG, GST-CagZ (negative controls for integrin binding) or GST-Inv397 (positive control) (30mg/1×106 cells) to integrin-deficient GE11 versus integrin-proficient GE11β cells by flow cytometry (n = 4). (C) Quantification of binding of purified GST-CagL or GST-CagL(RAD) (30mg/1×106 cells) to integrin-deficient GE11 versus integrin-proficient GE11β cells by flow cytometry (n = 6). Binding was analysed by anti-GST antibody (fold increase in binding versus GST, the values for binding of GST alone has been set to 1). (*, p<0.05; **, p<0.01; ***, p<0.001; students T-test). MF, mean fluorescence. (0.63 MB TIF) [file ppat.1000684.s005.tif]

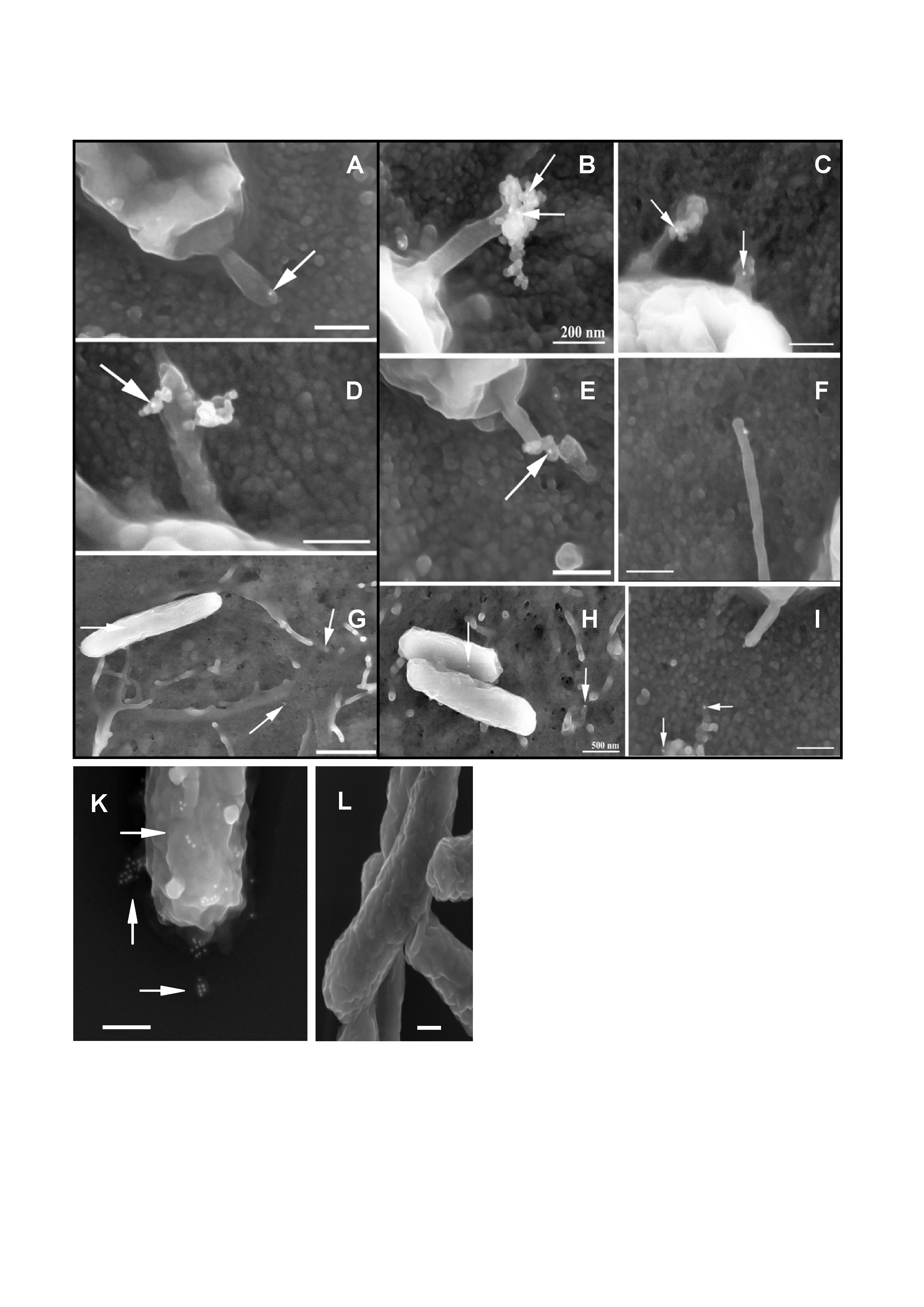

Supplement: Figure S3 — High resolution FESEM micro-graphs of AGS cells infected with H. pylori. (A–F) CagA labelling is repeatedly found on the tip region of the secretion pilus using immunogold labelled antibody AK273 (arrows) (G–I) P12ΔPAI strain showing only few gold-particles on the AGS cell surface (arrows in G and H); in control experiments with H. pylori-infected AGS cells incubated with the gold marker alone no labelling in the tip region of the secretion apparatus is detectable. The background labelling was comparable to the ΔPAI strain (arrows in I). (K–L) Localization of CagY protein on the OM and the cag-T4SS pilus of Hp 26695 wt, but not 26695ΔPAI using immunogold-labelled antiserum AK273 by FESEM. Bars represent 200 nm (A–F,K), 1 µm (G), 500 nm (H,I,L). (6.78 MB TIF) [file ppat.1000684.s006.tif]

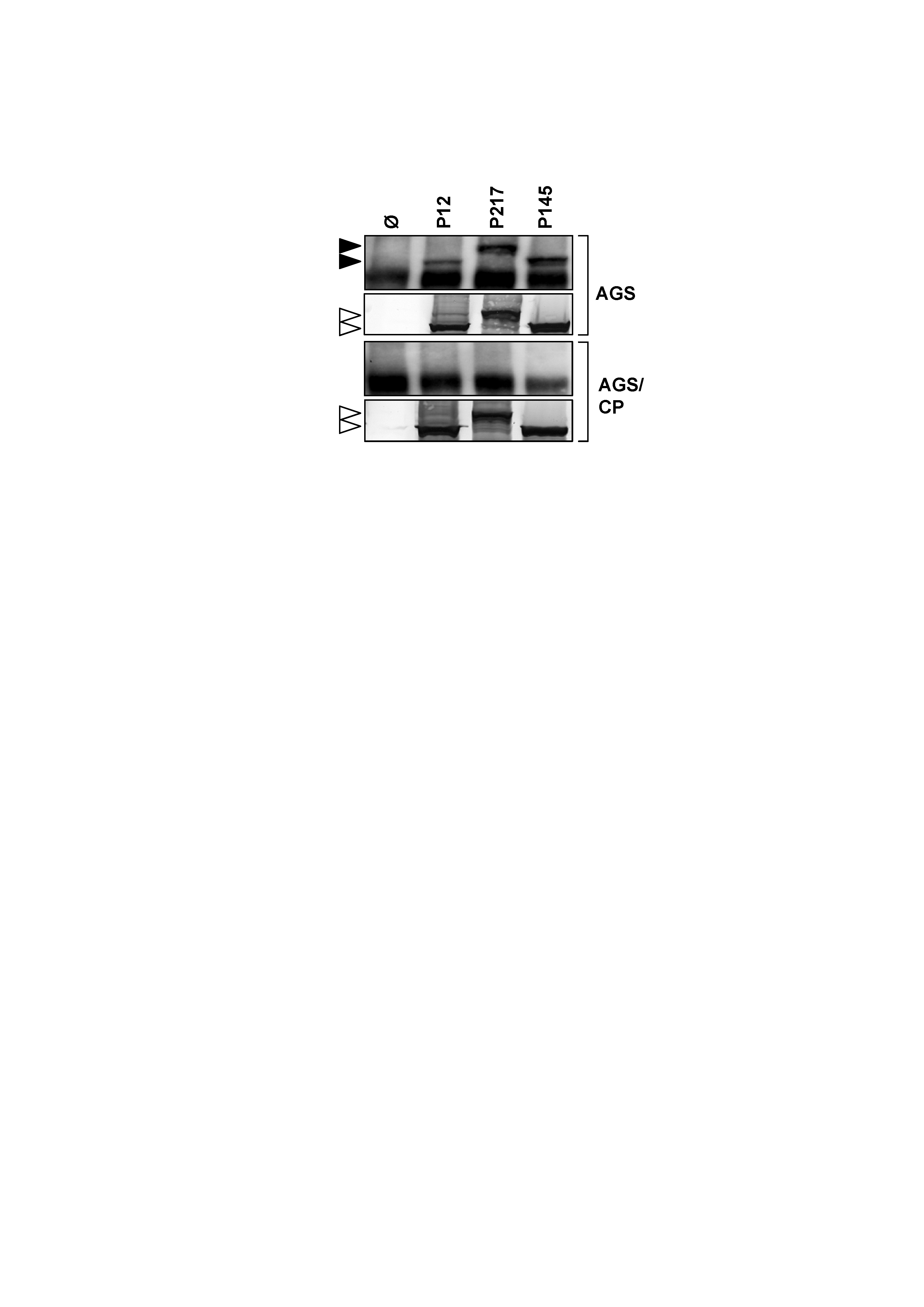

Supplement: Figure S4 — Pharmacological Iinhibitors interfering with CagA translocation in AGS epithelial cells. CagA translocation into AGS cells is inhibited by calpeptin (CP), an inhibitor of the Ca2+-dependent protease calpain. CP, calpain. Filled arrowheads mark CagAP-tyr, open arrowheads mark CagA bands. (0.79 MB TIF) [file ppat.1000684.s007.tif]
